# Editorial: Neurophysiological basis of the relationship between core stability and human movement: implications for sport and rehabilitation

**DOI:** 10.3389/fphys.2026.1916482

**Published:** 2026-07-14

**Authors:** Erika Zemková, Gerold Ebenbichler, Tomáš Malý, Magni Mohr

**Affiliations:** 1 Department of Biological and Medical Sciences, Faculty of Physical Education and Sport, Comenius University Bratislava, Bratislava, Slovakia; 2 Department of Physical Medicine, Rehabilitation & Occupational Medicine, Medical University of Vienna & Vienna General Hospital, Vienna, Austria; 3 Faculty of Physical Education and Sport, Charles University, Prague, Czechia; 4 Faculty of Health, University of the Faroe Islands, Tórshavn, Faroe Islands; 5 Department of Sports Science and Clinical Biomechanics, Sport and Health Sciences Cluster, Faculty of Health Sciences, University of Southern Denmark, Odense, Denmark

**Keywords:** balance function, core stability and strength, running, spine motion, walking, lifting task, physiological mechanisms, trunk rotation

Given the success of the previous Research Topic “A Physiologically-Based Approach to Study Different Types of Locomotion in Association with Core Performance” (Zemková et al., 2024) and the continuing advances in the field, a second edition of this Research Topic has been announced.

Good posture and strong core muscles are essential for most athletic movements, but also for daily life activities. Better neuromuscular control of postural and core stability contributes to more efficient functional movements (Zemková and Zapletalová, 2022). Among them, walking and running require lumbo-pelvic stability and mobility for efficient movement and high-level performance. It is especially important during extensive trunk motions while changing the direction of movement (Horníková and Zemková, 2024), an abrupt walk-to-run transition, or extreme uphill and downhill walking or running. For example, after a simulated soccer match in trained players, the degree of fatigue of the lumbar thoracic muscles was comparable to that of the ankle, knee and hip muscles, which can be perceived as directly involved in soccer movements (Fransson et al., 2018).

The core is equally important when rotating the trunk or lifting heavy loads in sports and in everyday activities. Such repetitive trunk loading over time may contribute to occurrence of back problems and lower limb injuries. Fatigue of the trunk muscles induced by excessive loading of the spine is one of the sources of back problems in athletes (Zemková et al., 2020). High training volume and repetitive motions are mainly responsible for the high prevalence rates. The most influential are biomechanical and physiological variations underlying the spine, though stress-related psychological factors should also be considered. The main neurophysiological risk factors leading to back problems in athletes are neuromuscular imbalance, increased muscle fatigability, muscle dysfunction and impaired motor control, while biomechanical risk factors include maladaptive spinal, spinopelvic and lower limb kinematics, side-to-side imbalances in axial strength and hip rotation range of motion, spinal overloading and deficits in movement pattern (Zemková et al., 2023).

In the general population, electromyographic instantaneous median frequency during cyclic lifting tasks was found to decrease significantly over time in paravertebral muscles but not in limb muscles (Bonato et al., 2003). Significant changes due to fatigue during the task were in angular displacements at the knee, hip, trunk, and elbow. These biomechanical changes were associated with increased peak torque and forces at the L4-L5 vertebral segment. Furthermore, lumbar muscle fatigue causes changes in the lumbar spinal curvature and this is functionally relevant in explaining the impaired ability to maintain balance after externally induced perturbations (Zemková et al., 2021). This emphasizes the importance for assessing both spinal posture and reactive balance control under fatigue to reveal their interrelations in young sedentary adults and predict any significant deterioration in later years.

Avoiding these unwanted effects requires a novel approach to studying the physiology of human movement in association with spinal motion and balance function. This may provide a basis for designing exercise programs specifically tailored for competitive athletes, the healthy general population, as well as those suffering from movement disorders. Although enhanced athletic performance is often attributed to improvements in neuromuscular function induced by sport-specific balance exercises, it could equally well be ascribed to improvements through general body conditioning exercises (Zemková and Kováčiková, 2023). Training programs incorporating general and sport-specific exercises that involve the use of postural and core muscles showed an improvement of body balance, back muscle strength, and muscular endurance (Zemková and Zapletalová, 2022). Core strength should be developed to the extent that allows race walkers and runners to optimize their performance (Zemková, 2025). Core function training contributes especially to improving change of direction speed (Horníková and Zemková, 2024). In addition, core strengthening and core stabilization exercises, alone or in combination with athlete training, contribute to the reduction of back pain in athletes (Zemková and Zapletalová, 2021). In sedentary employees, core muscle exercises reduce muscle fatigue caused by prolonged sitting (Amiri et al., 2025). Intervention studies with core muscle exercises, primarily targeting the erector spinae, significantly improve tensiomyography outcomes (Amiri et al., 2025).

So far, much effort has been devoted to investigating biomechanical and physiological variations of locomotion, including walking, running, swimming or hopping. However, a surprising evidence gap is in what extent core stability contributes to effective locomotor performance and a healthy back. There is also little research investigating the force-velocity-power characteristics of exercises involving the use of trunk muscles in athletes with different demands on core strength (Zemková, 2022). Methods reflecting the functional nature of core strength, along with measures of postural alignment and lateral lumbopelvic stability, should be used to assess training effectiveness. Recent perspective study highlights a comprehensive head-to-toe approach to assessing athletic locomotion (Zemková, 2025). Special emphasis should be placed on the role of postural and core stability in walking and running gait.

Therefore, studying neurophysiological mechanisms underlying core stability and strength with special reference to human motion is of great importance. A better understanding of the relationship between core stability and human movement may have implications for designing sports training and rehabilitation programs to enhance athletes’ performance and/or reduce their risk of back pain and lower limb injuries.

**Figure 1:**
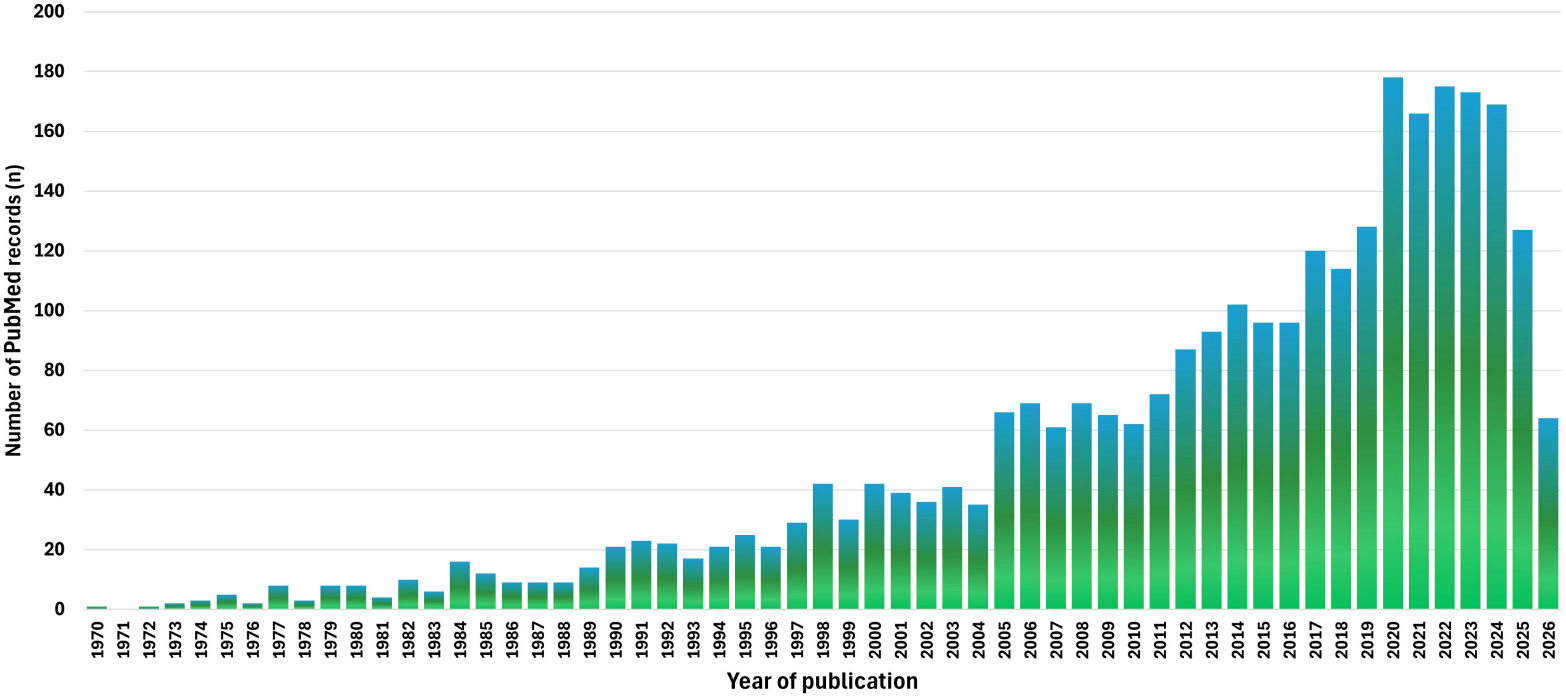
The results of a systematic search in PubMed according to the following Boolean search syntax: [“core stability” (All Fields)] OR [“core performance” (All Fields)] OR [“core strength” (All Fields)] AND [“human movement” (All Fields)] OR [“locomotion” (All Fields)] OR [“running” (All Fields)] OR [“walking” (All Fields)] AND [“neurophysiological mechanism” (All Fields)] OR [“physiological mechanism” (All Fields)].

The fact that this Research Topic is of great interest among researchers and practitioners is also evidenced by the exponentially increasing number of articles in the last decade. The **Figure 1** illustrates the results of a systematic search in PubMed according to the Boolean search syntax: “core stability” OR “core performance” OR “core strength” AND “human movement” OR “locomotion” OR “running” OR “walking” AND “neurophysiological mechanism” OR “physiological mechanism.” It includes 2639 times over 56 years.

Within this Research Topic, 26 articles were submitted, of which 8 were rejected and 18 accepted. This Research Topic presents a collection of eighteen papers that collectively demonstrate a shift from isolated strength-based approaches toward more integrated models incorporating proprioception, sensory processing, postural control, and sport-specific adaptation.

Several studies explore core function and postural regulation. For example, in perturbed postural tasks, core muscle co-activation has been shown to contribute to trunk stability and balance control during single-leg perturbations. Also, comparisons between elite martial artists and sprinters further demonstrated sport-specific neuromuscular strategies. Similarly, studies on young athletes revealed that soccer players displayed superior postural control compared with swimmers and non-athletes under increasingly difficult balance conditions, while attentional control in young female volleyball players was moderately associated with dynamic balance and injury-related biomechanics.

Balance and sensory integration were also examined in visually challenged conditions. TRX suspension training improved dynamic balance in surfers without visual input more effectively than traditional balance training. Moreover, in individuals with low vision and blindness, sensory reweighting strategies differed substantially.

Core strength and its functional significance represented another major theme. Although core strength is often assumed to enhance athletic performance through improved stability, mediation analysis found that core stability did not significantly mediate the relationship between core strength and jump performance. Likewise, trunk strength in elite swimmers showed limited associations with sprint swimming performance once confounding factors were controlled. Together, these findings suggest that efficient force transfer and intersegmental coordination may be more important than isolated strength measures alone.

In contrast, targeted interventions demonstrated meaningful benefits. Heavy-resistance core strength training improved upper-body power in junior swimmers and kayakers, while combining motor imagery with lumbar exercises significantly enhanced lumbar proprioception in elite swimmers. In performing arts students, one study focused on effects of musculoskeletal health education on dynamic spine function.

Rehabilitation and injury prevention were also strongly represented. A systematic review and meta-analysis on chronic non-specific low back pain showed that Pilates training was effective for pain reduction, whereas core resistance training improved functional status. Additionally, hamstring-focused research demonstrated that high-volume Nordic hamstring exercise protocols produced different architectural adaptations than low-volume protocols. Complementary findings further highlighted the relationship between isometric and eccentric hamstring strength.

Upper-extremity neuromuscular assessment was examined in elite baseball athletes, using isolated shoulder rotation tests and athletic shoulder tests. Overall, the findings point towards using multidimensional testing approaches in rehabilitation, performance monitoring, and return-to-sport decision-making for overhead athletes.

Finally, broader applications of exercise science were also addressed. For example, cluster analysis of physical fitness profiles of university students with the aim of applying individualized, data-driven approaches to optimize physical education programs and identify cardiometabolic risk. Also, the included review on neuromuscular adaptations to resistance training examplified how training status, genetics, age, and sex influence adaptation processes.

Collectively, the studies in this Research Topic reinforce the multidimensional nature of sport and exercise science. Across performance enhancement, rehabilitation, injury prevention, and health promotion, the findings consistently highlight that movement quality depends on the interaction of neuromuscular coordination, sensory integration, cognitive regulation, and sport-specific adaptation rather than isolated measures of strength or mobility. This growing integrative perspective will continue to guide future research and the development of individualized approaches to optimize both athletic performance and long-term musculoskeletal health.
